# Bonding Orthodontic Attachments to 3D-Printed Photosensitive Definitive Resin: An In Vitro Study

**DOI:** 10.3390/dj13080341

**Published:** 2025-07-24

**Authors:** Omaika Victoria Criollo-Barrios, Carlos Roberto Luna-Domínguez, Carlos Alberto Luna-Lara, Ricardo de Jesus Figueroa-López, Ronaldo Câmara Cozza, Jorge Humberto Luna-Domínguez

**Affiliations:** 1 Faculty of Dentistry, Autonomous University of Tamaulipas, Av. Universidad esq. con Blvd. Adolfo López Mateos s/n, Tampico C.P., Ciudad Victoria 89337, Mexico; omaika.criollo@uat.edu.mx (O.V.C.-B.); cldominguez@docentes.uat.edu.mx (C.R.L.-D.); cluna@docentes.uat.edu.mx (C.A.L.-L.); ricardo.figueroa@uat.edu.mx (R.d.J.F.-L.); 2 CEETEPS—State Center of Technological Education “Paula Souza”, Department of Mechanical Manufacturing, Av. Antônia Rosa Fioravante 804, Mauá 09390-120, SP, Brazil; ronaldo.cozza@fatec.sp.gov.br

**Keywords:** 3D printing resin, attachments, shear bond strength, surface treatment, universal adhesive

## Abstract

**Background/Objectives**: The increasing clinical integration of 3D-printed definitive resins requires a comprehensive understanding of their physicochemical properties and adhesive behavior. However, there is limited evidence regarding the optimal surface treatment and bonding strategies for clear aligner composite attachments on these materials. This study aimed to characterize a 3D-printed definitive resin, evaluate the effects of surface treatments on its surface topography, and compare the shear bond strength (SBS) of the bonded attachments using different adhesive systems, both before and after thermocycling. **Methods**: A total of 120 rectangular specimens were fabricated from a 3D printed dental resin (Crowntec^®^, SAREMCO Dental AG—Mexico City, Mexico). For physicochemical characterization, six samples underwent scanning electron microscopy/energy-dispersive spectroscopy, X-ray diffraction, and thermogravimetric analysis. To evaluate surface topography, 42 polished specimens were assigned to three groups: untreated (control), etched with 4% hydrofluoric acid (HFA), or sandblasted with 50 µm Al_2_O_3_ (AA). Each group was subdivided for SEM observation and surface roughness (*Ra*) measurement. For SBS testing, 72 additional samples received the same surface treatments and were further subdivided according to the adhesive system: Transbond™ XT Primer (TXT) or Single Bond Universal (SBU). **Results**: The AA group showed the highest *Ra* (2.21 ± 0.30 µm), followed by HFA (0.81 ± 0.20 µm) and control (0.07 ± 0.30 µm) (*p* < 0.001). The highest SBS was observed in the AA + SBU group, followed by AA + TXT. **Conclusions**: Sandblasting with Al_2_O_3_ particles, combined with a universal adhesive, significantly improved bond strength, suggesting a viable protocol for 3D printed definitive composites in aligner attachment applications.

## 1. Introduction

Contemporary dentistry has experienced a profound digital transformation through the integration of CAD/CAM systems that employ subtractive and additive manufacturing methods for the fabrication of prosthetic restorations [1]. Dental milling machines have been extensively validated in the literature for their ability to fabricate ceramic, resin, and hybrid restorations with excellent replication of tooth morphology, dimensional accuracy, internal fit, and efficient manufacturing times [1,2,3]. As a result, they represent a reliable and widely adopted method for producing a variety of dental prostheses, including crowns, bridges, veneers, inlays, and onlays [2,3]. Despite these advantages, subtractive manufacturing inherently involves considerable material wastage (up to 70% of the original milling block) and constraints on geometric complexity due to the limited accessibility of milling burs [4,5].

On the contrary, additive manufacturing (AM) by VAT photopolymerization enables the layer-by-layer construction of complex structures by selectively curing liquid resin through targeted light-activated polymerization, guided by a digital model [6]. This approach allows a more efficient use of materials and substantially reduces production times [6,7]. Recent investigations have shown that the marginal and internal fit of 3D-printed crowns can be comparable to those produced by subtractive methods, thus meeting clinical standards for precise adaptation [8,9,10].

Within this context, definitive photosensitive composite resins have emerged as an innovative alternative to 3D printed permanent restorations, encompassing crowns, inlays, veneers, and even prosthetic teeth [11]. Among these resins, Crowntec^®^ (SAREMCO Dental AG—Mexico City, Mexico) is notable for comprising a methacrylate-based monomer matrix reinforced with inorganic fillers (30–50% by weight). This formulation confers more than 150 MPa flexural strength, dimensional stability after photopolymerization, favorable optical characteristics, and clinically acceptable esthetics, largely attributable to its translucency and color stability after artificial ageing [12,13,14,15].

In parallel, the growing number of adult patients seeking orthodontic treatment, many of whom have preexisting prosthetic restorations, requires bonding orthodontic device—such as buttons, brackets, or auxiliary attachments—to natural tooth surfaces and a variety of restorative substrates (CAD/CAM ceramics, porcelains, metals, and more recently, 3D printed resins) [16,17,18,19]. In the specific context of clear aligner therapy, the secure adhesion of attachments to these substrates is critical to achieve precise control over tooth movement [20,21,22,23].

Given this scenario, a comprehensive understanding of the chemical composition and microstructure of 3D-printed resins is indispensable to select surface conditioning strategies that favor mechanical microretention and stable chemical bonding. In materials with high glass content (e.g., silica), surfaces are commonly etched with hydrofluoric acid (HFA) and subsequently treated with a silane primer, creating microretentive features in the vitreous phase and siloxane bonds with the adhesive resin. In contrast, for predominantly organic matrices or those containing metal oxides, airborne particle abrasion with aluminum oxide (Al_2_O_3_) is commonly used followed by the application of primers incorporating functional monomers such as 10-methacryloyloxydecyl dihydrogen phosphate (MDP) [11,24,25,26].

Universal adhesives offer a promising approach to address the heterogeneous nature of these substrates. By incorporating multiple functional monomers (MDP, silane, and hydrophilic and hydrophobic acrylates) into a single formulation, these products facilitate effective bonding to a wide variety of materials, including enamel, dentin, metals, ceramics, and resins, thus streamlining clinical procedures compared to conventional adhesives [27,28,29]. In fact, several investigations report that universal adhesives can achieve bond strengths comparable to or surpassing those of conventional orthodontic bonding systems, particularly in ceramic materials such as lithium disilicate and zirconia [30,31,32].

Despite recent advances, evidence on adhesive protocols specifically developed for definitive 3D-printed resins remains limited. To the best of our knowledge, this study stands as one of the pioneering efforts to integrate an extensive physicochemical characterization, including microstructural, elemental, and thermal aspects—of a definitive 3D printed composite resin designed for long-term intraoral applications with a mechanism-driven evaluation of bonding methodologies for clear aligner attachments. By explicitly associating the material’s hybrid organic–inorganic architecture with two standard surface-conditioning techniques (airborne particle abrasion and hydrofluoric acid etching) and two clinically available adhesive systems (a universal adhesive and a conventional orthodontic primer), this study transcends the empirical protocols previously reported. Given that clear aligner therapy is increasingly dependent on reliable attachment bonding to permanent 3D printed crowns and bridges, the development of quantitative, clinically oriented guidance for these novel restorative substrates is both innovative and urgently required. This study aimed to characterize the physicochemical properties of a definitive photosensitive 3D-printed resin, evaluate the effects of surface treatments (4% hydrofluoric acid etching and airborne-particle abrasion with 50 µm Al_2_O_3_) on its surface topography, and assess the bond strength of orthodontic attachments using two adhesive systems (3M^TM^ Single Bond Universal—Mexico City, Mexico and Primer 3M^TM^ Transbond^TM^ XT—Mexico City, Mexico) under thermocycling. The findings are expected to inform the development of reliable and clinically effective bonding protocols for aligner-based orthodontic treatment that involves 3D printed permanent restorations.

## 2. Materials and Methods

### 2.1. Materials Used In Vitro Study

The materials used in this in vitro study are shown in Table 1: 3D printed composite resins, bonding agents and composite resin.

### 2.2. Preparation of 3D Printed Samples

**Figure 1:**
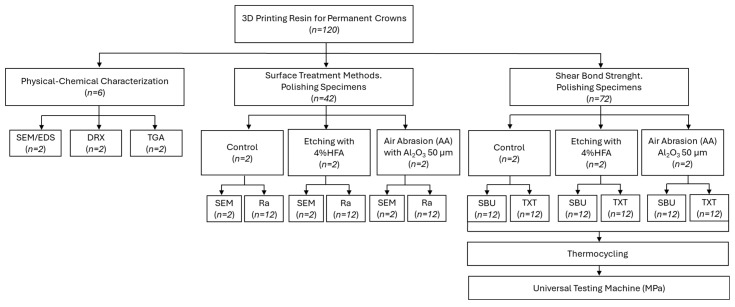
Experiment design.

A total of 120 rectangular specimens (10 mm × 5 mm × 1 mm) were designed using Computer-Aided Design software (Fusion 360, Autodesk Inc.—Mexico City, Mexico). The files were exported in standard tessellation language (STL) format and subsequently processed using 3D Sprint^TM^ software (3D Systems, Inc.—Mexico City, Mexico), arranging the samples in groups of ten at a 0° orientation relative to the build platform [33,34]. A 3D printed composite resin for definitive restoration (Crowntec, SAREMCO Dental AG—Mexico City, Mexico) was used for specimen fabrication. The resin was homogenized in a dedicated mixer (LC-3DMixer, 3D Systems, Inc.—Mexico) for 5 min, then transferred to the resin tray of a digital light processing (DLP) 3D Printer (NextDent 5100, 3D Systems, Inc.—Mexico). Printing was initiated by transferring the STL files via USB interface. After that, the printed specimens were thoroughly rinsed in 99% ethyl alcohol using an ultrasonic bath in combination with a soft bristle brush to remove uncured resin residues. Post-curing was carried out in a UV polymerization chamber (LC-3DPrint Box, 3D Systems, Inc.—Mexico) for 30 min according to the manufacturer’s instructions. Subsequently, all samples were trimmed with a diamond disc under water cooling and allocated for further analysis, as illustrated in Figure 1.

### 2.3. Physical–Chemical Characterization

Six unpolished Crowntec resin specimens were assigned to three groups (*n* = 2 per group) for physicochemical characterization using complementary analytical techniques.

#### 2.3.1. Scanning Electron Microscopy and Energy-Dispersive X-Ray Spectroscopy (SEM/EDS)

Specimens were mounted on aluminum stubs and examined using a field-emission scanning electron microscope (JSM-7401F, JEOL Ltd.—Tokyo, Japan) equipped with an energy-dispersive X-ray spectroscopy detector (EDAX, AMETEK Inc.—Mahwah, NJ, USA). SEM imaging and elemental EDS analysis were performed at an accelerating voltage of 15 kV under high vacuum.

#### 2.3.2. X-Ray Diffraction (XRD)

Crystalline phase analysis was performed using PANalytical X’Pert Pro MPD—Materials Powder Diffractometer (Malvern Panalytical Ltd.—Almelo, The Netherlands). Specimens were placed on a standard sample holder and diffraction patterns were recorded in a 2*θ* range of 5° to 10°, with a step size of 0.016° and a counting time of 300 s per step, using X’Celerator Solid State Detector (Malvern Panalytical Ltd.—Almelo, The Netherlands).

#### 2.3.3. Thermogravimetric Analysis (TGA)

Thermal stability and compositional analysis were performed using a simultaneous TGA/DSC analysis (Universal V4.5A Analyzer, TA Instruments—New Castle, DE, USA). The specimens were heated from 25 °C to 800 °C at a constant rate of 10 °C/min in an air atmosphere and mass loss was continuously recorded to evaluate the organic/inorganic content.

### 2.4. Surface Treatment Methods—Surface Morphology and Surface Roughness (Ra)

A total of 42 Crowntec resin-fabricated specimens were polished using a porcelain polishing system (DIATECH, COLTENE-Whaledent AG Inc.—Altstätten, Switzerland) and randomly assigned to three experimental groups according to the surface treatment applied: (1) no treatment (control); (2) chemical etching with 4% HFA gel (BISCO Inc.—Schaumburg, IL, USA) for 60 s [35,36]; and (3) airborne particle abrasion (AA) with 50 μm Al_2_O_3_ particles for 10 s at 0.2 MPa pressure and a working distance of 10 mm [13,37,38]. After surface treatment, all samples were rinsed with sterile distilled water for 5 s and dried with oil-free compressed air.

Each surface treatment group was subdivided into two types of analysis: quantitative surface roughness measurement (*Ra*) and qualitative surface morphology evaluation. For surface roughness analysis, a priori, the sample size calculation was performed using G*Power 3.1 software (Heinrich Heine University—Düsseldorf, Germany). Based on a statistical power of 0.85, an alpha error probability of 0.05, and an effect size of 0.70 (Cohen’s f), the required sample size was determined to be twelve specimens per group (*n* = 12). A contact profilometer (Portable Surface Roughness Tester Surftest SJ-410 Series, Mitutoyo America Corporation, Aurora, IL, USA) was used to measure surface roughness (*Ra*) according to Standard ISO 21920-2:2021 [39].

For qualitative evaluation of surface morphology, two additional specimens per group (*n* = 2) were subjected to scanning electron microscopy (SEM). Before imaging, the samples were inspected under a stereomicroscope, mounted on aluminum stubs, sprayed with gold (SCD 050 Sputter Coater—BalTec AG, Pfäffikon, Switzerland), and examined using a field-emission SEM (JSM-7401F, JEOL Ltd.—Tokyo, Japan) operated at an acceleration voltage of 15 kV.

### 2.5. Shear Bond Strength (SBS) Test

#### 2.5.1. Attachment Fabrication

Rectangular attachments (5.0 mm × 2.0 mm × 2.0 mm) were digitally designed using computer-aided design software (Fusion 360, Autodesk Inc.—Mexico) and printed using a model resin. From the printed models, custom matrices were made with 0.020″ thermoplastic sheets (Zendura^TM^ FLX, Bay Materials LLC—Fremont, CA, USA) using a pressure molding thermoforming unit (MINISTAR S^®^, SCHEU-DENTAL GmbH, Iserlohn, Germany), following the manufacturer’s recommended settings. These matrices were used to standardize the shape and dimensions of composite attachments fabricated from nanohybrid composite resin (Filtek^TM^ Z350 XT, 3M^TM^ ESPE^TM^—Mexico City, Mexico).

#### 2.5.2. Preparation and Grouping of Specimens

Seventy-two rectangular specimens (10 mm × 5 mm × 1 mm) of the definitive Crowntec resin printed in 3D were polished using a porcelain polishing system (DIATECH, COLTENE-Whaledent AG Inc.—Altstätten, Switzerland). The samples were randomly assigned to three surface treatment groups (*n* = 24 per group): (1) no treatment (control), and (2) etching with 4% HFA (BISCO Inc.—Schaumburg, IL, USA) for 60 s and (3) AA with 50 μm Al_2_O_3_ particles for 10 s at 0.2 MPa and 10 mm distance. All samples were rinsed with sterile distilled water for 5 s and air dried with oil-free compressed air.

Each surface treatment group was further divided into two adhesive subgroups (*n* = 12) according to the bonding protocol:

- TXT—Transbond^TM^ XT Light Cure Adhesive Primer (3M^TM^ Unitek^TM^—Mexico City, Mexico).
- SBU—Single Bond^TM^ Universal Adhesive (3M^TM^ ESPE^TM^—Mexico City, Mexico).

In the TXT subgroup, a thin layer of primer was applied directly to the printed resin surface without precuring, as per the manufacturer’s instructions. The composite attachment was immediately positioned and light-cured for 10 s on both the mesial and distal surfaces. In the SBU subgroup, the adhesive was actively applied to the bonding surface for 20 s, followed by gentle air drying for 5 s and light cure for 10 s prior to attachment placement. The composite attachments were then placed and light cured in the same manner.

#### 2.5.3. Thermocycling and Mechanical Testing

All bonded specimens were subjected to artificial aging by thermocycling for 500 cycles between 5 °C and 55 °C, with a dwell time of 30 s and a transfer interval of 5 s [25,40,41]. This thermocycling regimen was selected to simulate the early stage thermal fatigue experienced by orthodontic attachments under clinical conditions. According to Standard ISO/TS 11405:2015 [42], 500 cycles represent the standard in vitro protocol to assess the initial durability of the bond. Following thermocycling, each specimen was embedded in autopolymerizing acrylic resin blocks (Líquido Normal Nictone 250 mL Monómero, MDC Dental—Zapopan, Jalisco, Mexico), with the bonded surface orientated parallel to the direction of the applied force.

The shear bond strength was measured using a universal testing machine (Alliance RT/30, MTS Systems Corporation, Eden Prairie, MN, USA) equipped with a chisel-edge blade. The load was applied at a crosshead speed of 1 mm/min until debonding occurred. The maximum force required to dislodge each attachment was recorded in Newtons (N) and converted to shear bond strength (MPa) by dividing by the bonded surface area.

### 2.6. Statistical Analysis

For the *Ra* data, one-way analysis of variance (ANOVA) was used to compare surface roughness between the three treatment groups. For SBS, a two-way ANOVA was performed to evaluate the main effects of surface treatment and adhesive system, as well as their interaction on bond strength. Post hoc pairwise comparisons were performed using the Games–Howell test (*p* < 0.05) to address unequal variances and sample sizes. All statistical analyses were performed with SPSS 25 software (IBM Co., New York, NY, USA), and statistical significance was established at α = 0.05.

## 3. Results

### 3.1. Physical–Chemical Characterization

#### 3.1.1. Surface Morphology and Elemental Composition (SEM/EDS)

**Figure 2:**
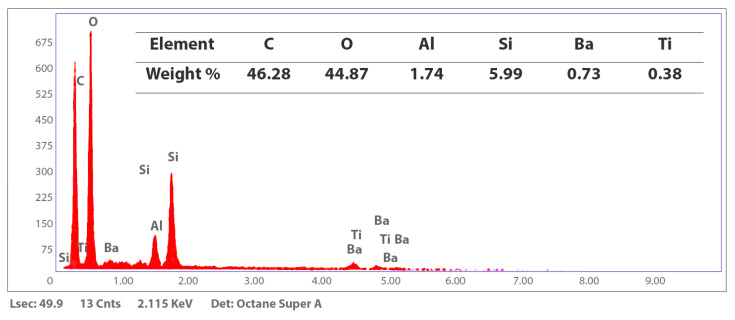
Energy-dispersive X-ray spectroscopy (EDS) spectrum of the Crowntec resin surface. Dominant peaks for carbon and oxygen reflect the polymeric organic matrix, while lower intensity peaks for silicon, aluminum, barium, and titanium indicate the presence of dispersed inorganic fillers.

The elemental composition of the unpolished Crowntec resin surface was evaluated using energy-dispersive X-ray spectroscopy (EDS), revealing a matrix predominantly composed of carbon (46.28 wt%) and oxygen (44.87 wt%), with minor concentrations of silicon (5.99 wt%), aluminum (1.74 wt%), barium (0.73 wt%) and titanium (0.38 wt%) (Figure 2). The corresponding EDS spectrum exhibited intense peaks for carbon and oxygen, consistent with the polymeric organic matrix of the material. Peaks for silicon, aluminum, barium, and titanium were present at lower intensities, reflecting their role as part of the resin’s inorganic filler phase.

#### 3.1.2. Crystalline Structure Analysis (XRD)

**Figure 3:**
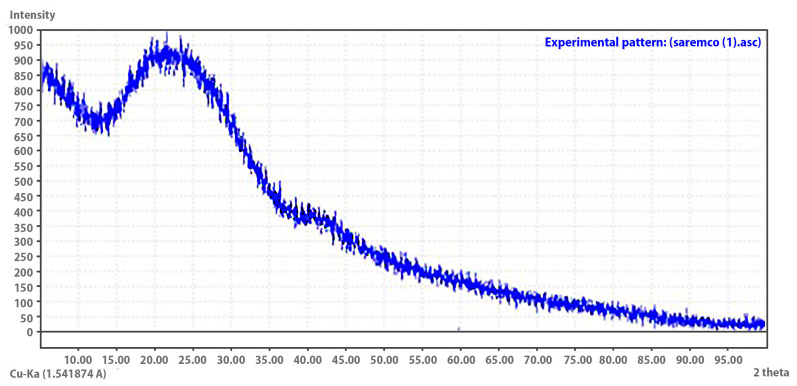
X-ray diffraction (XRD) pattern of the 3D printed Crowntec resin. The broad diffuse halo between 10° and 35° (2*θ*) without distinct Bragg peaks indicates an amorphous structure characteristic of polymer-based restorative materials.

The X-ray diffraction (XRD) pattern of the 3D-printed Crowntec resin exhibited a broad diffuse hump between approximately 10° and 35° (2*θ*), without the presence of sharp or well-defined Bragg peaks (Figure 3). This pattern is indicative of an amorphous structure, suggesting the absence of long-range atomic order or crystalline phases detectable by XRD. The lack of diffraction peaks confirms that the resin matrix is predominantly amorphous, which is consistent with the expected molecular arrangement of cross-linked, light-cured polymeric systems used in photopolymerizable dental materials.

#### 3.1.3. Thermal Stability and Composition (TGA)

**Figure 4:**
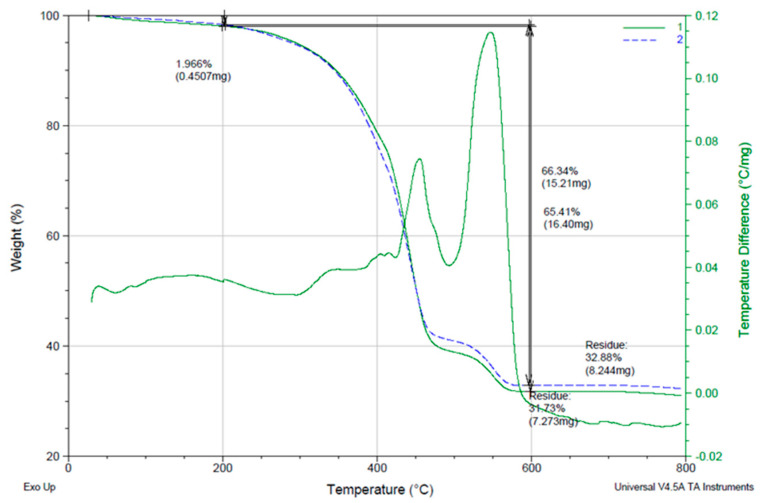
Thermogravimetric analysis (TGA) curve of the 3D-Printed Crowntec resin.

Thermogravimetric analysis (TGA) of Crowntec resin 3D printed revealed a major mass loss event of approximately 66.34% occurring between 200 °C and 600 °C, corresponding to thermal degradation of the organic polymer matrix (Figure 4). A minimal initial weight loss of 1.97% was observed below 200 °C, likely associated with the evaporation of adsorbed moisture or residual volatiles.

A major weight loss of ≈66% occurred between 200 °C and 600 °C, corresponding to the degradation of the organic matrix. A stable inorganic residue of ≈33% remained above 600 °C, confirming the hybrid organic-inorganic composition of the material.

Beyond 600 °C, the mass stabilized, with a final residual weight of 32.88%, indicative of a non-volatile inorganic filler fraction. The presence of this stable residue supports the hybrid nature of the material, comprising a polymerizable organic matrix reinforced by inorganic components, as previously suggested by EDS and XRD findings. The onset of significant degradation above 200 °C confirms that the material exhibits adequate thermal stability for intraoral conditions and standard processing protocols.

### 3.2. Surface Treatment Methods

#### 3.2.1. Surface Morphology (SEM Analysis)

**Figure 5:**
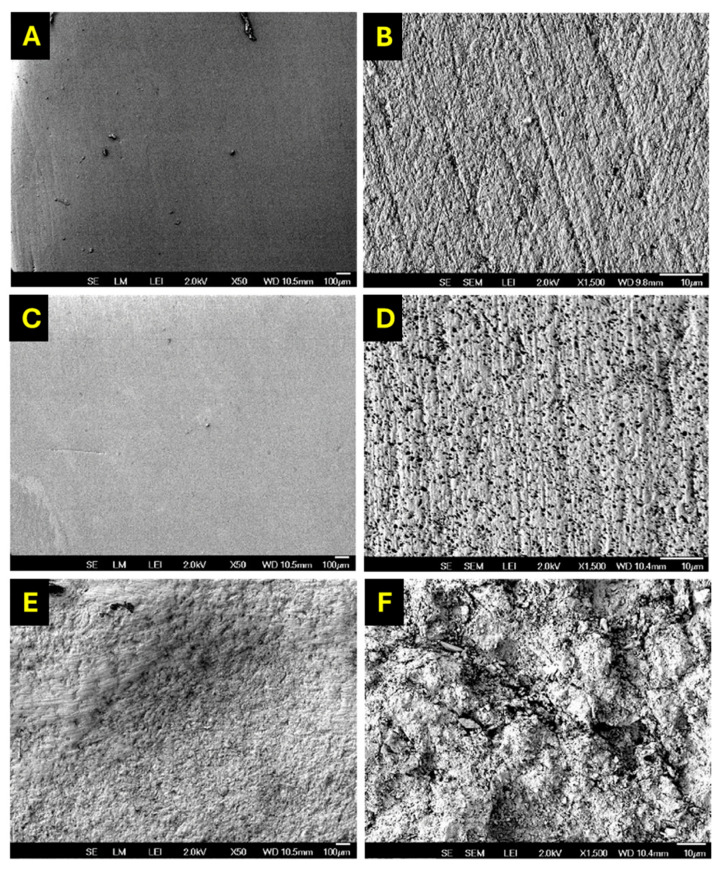
SEM micrographs of Crowntec^®^ resin specimens after different surface treatments—topographical differences observed for each surface conditioning protocol: (**A**) control—50×, (**B**) control—1500×, (**C**) etching 4% HFA—50×, (**D**) etching 4% HFA—1500×, (**E**) AA with 50 µm Al_2_O_3_—50× and (**F**) AA with 50 µm Al_2_O_3_—1500×.

Scanning electron microscopy (SEM) analysis at 50× and 1500× magnifications revealed distinct surface features among the three conditioning protocols applied to the Crowntec resin specimens (Figure 5A–F). The control group (no treatment) exhibited a smooth and homogeneous surface with minimal irregularities and low surface roughness, indicative of limited micromechanical retention capacity. On the contrary, specimens treated with 4% HFA displayed finely distributed microporosities, suggesting superficial etching likely caused by selective dissolution of the inorganic filler phase. This microtextured pattern may facilitate moderate adhesive penetration without compromising structural integrity. The most pronounced surface modification was observed after AA with 50 µm Al_2_O_3_ particles, which produced a highly irregular topography characterized by deep craters, grooves, and surface roughening. This morphological alteration significantly increased the available bonding area and provided well-defined microretentive features that were conducive to enhanced mechanical interlocking with adhesive systems.

#### 3.2.2. Surface Roughness Analysis (*Ra* Values)

**Figure 6:**
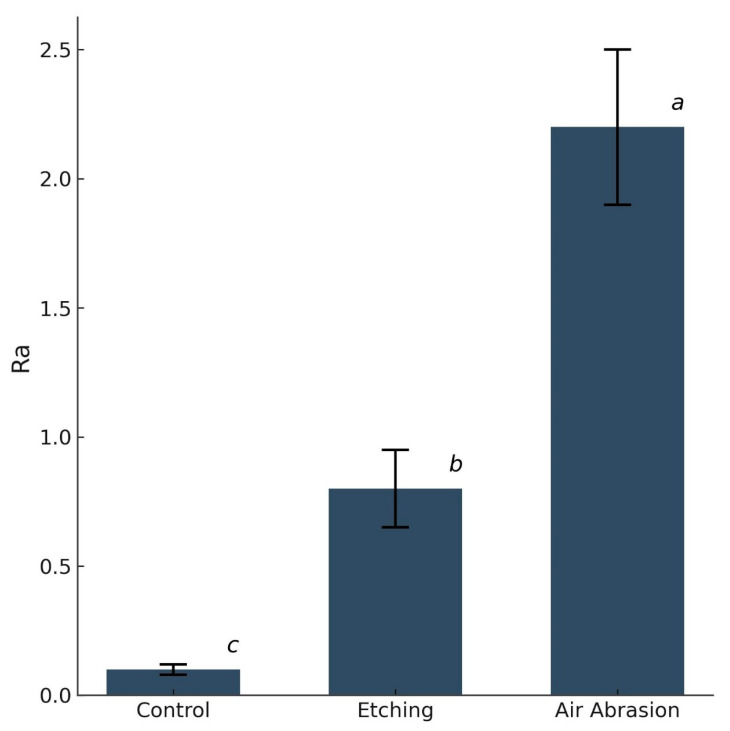
Mean surface roughness (*Ra*, µm) of Crowntec resin specimens after air abrasion, hydrofluoric acid etching, and no surface treatment (control). Error bars represent standard deviation. Statistically significant differences identified between all groups (one-way ANOVA, *p* < 0.001), with distinct superscript letters indicating significance in post hoc comparisons (Games–Howell, *p* < 0.05).

Quantitative surface roughness measurements revealed significant differences among surface treatment groups (Figure 6). The AA group exhibited the highest mean roughness (*Ra* = 2.21 ± 0.30 µm), followed by the etching group treated with 4% HFA (*Ra* = 0.81 ± 0.20 µm). The control group (without surface treatment) showed the lowest roughness values (*Ra* = 0.07 ± 0.03 µm).

Statistical analysis confirmed that the differences in *Ra* between all groups were statistically significant (*p* < 0.05). Post hoc comparisons indicated that each group differed significantly from the others, as denoted by distinct superscript letters.

### 3.3. Shear Bond Strength (SBS) Analysis

A two-way ANOVA demonstrated statistically significant main effects for both surface treatment (*F* = 125.41; *p* < 0.001) and adhesive system (*F* = 74.35; *p* < 0.001), as well as a significant interaction between the two factors (*F* = 4.19; *p* = 0.019) (Table 2).

**Figure 7:**
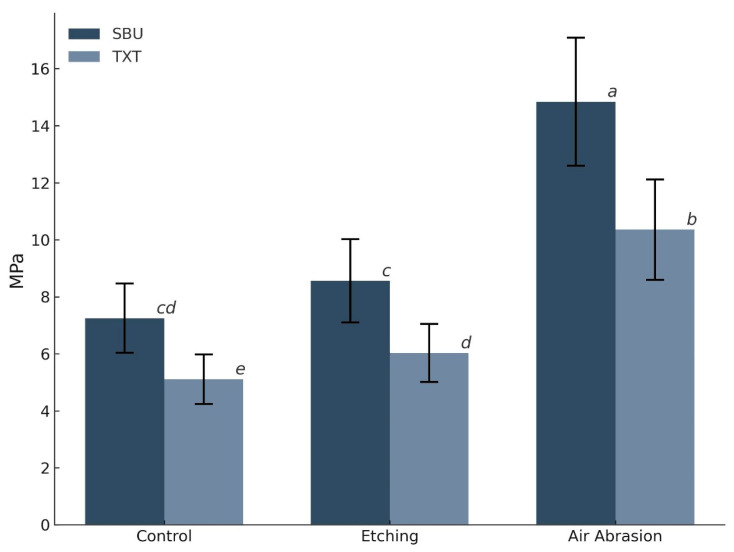
Shear bond strength (MPa) of Crowntec resin specimens as a function of surface treatment and adhesive system. The error bars represent the standard deviation. Different superscript letters denote statistically significant differences between groups (*p* < 0.05, Games–Howell post hoc test).

Among all groups, airborne particle abrasion yielded the highest bond strength values, with SBU achieving 14.84 ± 2.24 MPa and TXT achieving 10.36 ± 1.76 MPa. This was followed by the HFA-etched group, with SBS values of 8.56 ± 1.46 MPa for SBU and 6.03 ± 1.02 MPa for TXT. The control group exhibited the lowest bond strength: 7.25 ± 1.21 MPa for SBU and 5.11 ± 0.87 MPa for TXT (Figure 7).

Air abrasion produced the highest bond strengths for both adhesives, with SBU consistently outperforming TXT under all conditions.

Pairwise comparisons using the Games–Howell test revealed that air abrasion produced significantly higher SBS values than both etching and control treatments in adhesives (*p* < 0.001). SBU consistently outperformed TXT within each surface treatment group, with the greatest difference observed under air abrasion conditions (Δ = 4.48 MPa; *p* < 0.001). However, no statistically significant differences were observed between SBU in the control vs. etching groups (*p* = 0.545), or between TXT in the same groups (*p* = 0.759 and 0.999), reflecting a similarly moderate bond strength under these conditions (Table 3).

Failure mode analysis indicated that adhesive failure was the predominant mode in all groups, occurring in 80% of samples, regardless of surface treatment or adhesive type.

## 4. Discussion

This study evaluated the physicochemical characteristics and bonding performance of a definitive 3D printed resin (Crowntec) subjected to different surface treatments and adhesive systems. The findings confirm that the material exhibits a hybrid composition—primarily a cross-linked methacrylate-based polymer matrix reinforced with amorphous inorganic fillers—typical of nanohybrid or microfilled composite resins used for permanent dental restorations. These properties have critical implications for surface conditioning protocols and adhesive selection, particularly when bonding orthodontic attachments to 3D printed substrates.

Energy-dispersive X-ray spectroscopy (EDS) analysis of the 3D-printed definitive resin (Crowntec) revealed a predominantly organic composition, with carbon and oxygen comprising approximately 46% and 45% by weight, respectively, along with minor amounts of silicon, aluminum, barium, and titanium. This elemental profile is characteristic of hybrid methacrylate-based composites reinforced with barium–aluminum silicate glass fillers and radiopaque oxides such as titania, consistent with other studies evaluating DLP-printed materials for definitive restorations [12,13,14,38]. These findings align with previous reports by Donmez et al. [12] and Çakmak et al. [10], who documented similar filler compositions in printed composite crowns intended for long-term intraoral use. Complementary structural analysis by X-ray diffraction (XRD) demonstrated a broad amorphous halo between 10° and 35° (2*θ*), with no discernible Bragg peaks, confirming the lack of long-range crystalline order. This amorphous pattern is typical of crosslinked photopolymerized resin matrices and has been similarly observed in CAD/CAM and 3D-printed hybrid composites evaluated by Di Fiore et al. [14] and Mao et al. [15], who emphasized the role of filler distribution and polymer network structure in determining both mechanical and adhesive performance. Furthermore, thermogravimetric analysis (TGA) revealed a mass loss of approximately 66% between 200 °C and 600 °C, associated with the decomposition of the organic matrix, and a stable inorganic residue of 32.88% beyond 600 °C. These results are in agreement with previous TGA findings in 3D printed definitive resins, confirming the manufacturer’s reported filler content (30–50 wt%) and supporting the hybrid classification of the material [13,14,38]. Taken together, the EDS, XRD, and TGA data demonstrate that Crowntec behaves as a hybrid restorative composite featuring a highly filled amorphous polymer matrix interspersed with glass-based inorganic particles.

A predominantly polymer-based matrix requires surface treatments that enhance micromechanical retention, which is critical to achieving durable adhesion. In the present study, airborne particle abrasion using 50 μm Al_2_O_3_ at 0.2 MPa and a working distance of 10 mm resulted in the highest surface roughness and, correspondingly, the highest shear bond strength (SBS). These findings are consistent with previous research that shows that air abrasion creates an irregular topography with craters and grooves that increase the effective bonding area and provide undercuts for adhesive infiltration [13,15,36]. Studies by Kang et al. [13], Mao et al. [15] and Ersöz et al. [38] have further confirmed that this surface morphology promotes improved bond durability in definitive 3D-printed resins such as Crowntec, particularly under thermocycling. Di Fiore et al. [14] also reported that this treatment increases surface free energy, facilitating uniform adhesive wetting. Importantly, when the abrasion parameters used in this study are applied, no damage is induced to the mechanical integrity of the 3D printed resin. This is supported by previous studies that demonstrate that such settings do not generate microcracks or internal defects, nor do they reduce flexural strength or bonding performance in polymer-based printed composites [14,15,38]. Collectively, the evidence supports airborne particle abrasion as a safe and highly effective surface conditioning method for clinical use with 3D printed definitive resins. The higher standard deviation observed in the air abrasion group can be explained by the multifactorial and inherently stochastic nature of the particle projection. Although the nominal parameters were maintained constant, minor clinical variations in jet angulation, dynamic distance, and local exposure of inorganic fillers may result in heterogeneous surface topographies across specimens. Each particle impact generates variable-depth craters and ridges, producing surfaces that combine highly retentive areas with relatively smooth regions. This heterogeneity increases both the surface roughness (*Ra*) and the dispersion of the adhesive strength values. Controlled investigations have shown that minor adjustments, as minor as 3°–5° in angulation or 2–3 mm in distance, can modify *Ra* by up to 40% and result in a double increase in the standard deviation of SBS compared to chemically conditioned surfaces [13,43,44]. These findings substantiate the notion that while air abrasion is effective in establishing micromechanical retention, it inherently exhibits variability. Therefore, the use of 3D printed positioning guides to standardize angulation (90°) and distance (10 mm) is a possible alternative to reduce dispersion while preserving bonding performance. In the case of etching with 4% hydrofluoric acid (HFA), it produced a measurable increase in surface roughness and bond strength compared to the control group; however, its overall effectiveness was still lower than that achieved by air abrasion. These findings can be attributed to the selective action of HFA in silica-based fillers; the acid partially dissolves exposed glass particles, creating microporosities that increase surface energy and promote adhesive wetting [26,32]. However, as noted by Çakmak et al. [10] and Donmez et al. [12], the reaction remains inherently limited in 3D printed resin specimens such as Crowntec, whose formulation contains a reduced fraction of etchable glass and incorporates oxides such as Al_2_O_3_ and TiO_2_ that are less susceptible to acid attack [10,11,26].

The adhesive behavior observed in this study was affected by the bonding system used, reflecting the chemical composition of the substrate. Our research indicates that the latest generation universal adhesive (Single Bond Universal) outperformed a traditional orthodontic primer (Transbond XT), regardless of whether the specimens had undergone surface conditioning. This result can be attributed to the presence of functional molecules within the universal adhesive, specifically 10-methacryloyloxydecyl dihydrogen phosphate (10-MDP) and the silane coupling agent, both of which promote chemical interaction with the inorganic components of the 3D-printed resin. The silane coupling agent enhances adhesion by forming siloxane bonds with exposed silica (SiO_2_) particles following acid etching, thereby strengthening the chemical integration with the resin’s filler phase [4,13,28]. In parallel, the phosphate functional group of 10-MDP has a high affinity for metal oxides such as alumina and titania, enabling additional chemical bonding at the adhesive–substrate interface [28,30,38]. In comparison, a typical primer, which is largely composed of methacrylic resin, relies predominantly on micromechanical retention without providing this improved chemical connectivity. This limitation is particularly evident on smooth, untreated surfaces, where bond strength tends to be significantly reduced—a finding that has also been reported in previous studies evaluating the performance of conventional primers on 3D-printed resins [13,38,43]. The superiority of combining air abrasion with a universal adhesive thus reflects this synergy: the optimal surface roughness achieved by air abrasion, together with the chemistry of a universal adhesive that can effectively bond to the printed resin, not only ensures a bond strength above the threshold required to prevent debonding during treatment, but also supports a controlled and predictable removal of the attachment at the end of therapy. Importantly, recent in vitro evidence suggests that the use of fine rotary instrumentation, rather than conventional mechanical debonding techniques, allows selective removal of composite attachments without inducing cohesive failure on the 3D-printed resin substrate [45]. This minimally invasive approach preserves the structural and aesthetic integrity of the definitive restoration, which is particularly relevant in adult patients who undergo interdisciplinary orthodontic-prosthetic treatment where the prosthetic element must remain intact after orthodontic therapy.

This in vitro study did not account for all intraoral conditions like chewing forces, moisture levels, or enzymatic breakdown, which might influence the long-term performance of adhesives. Consequently, future studies should incorporate in vivo research to evaluate the clinical longevity of bonded attachments in 3D printed definitive restorations that experience functional stresses and thermal cycling over time. It is also essential to investigate how attachment removal affects the surface condition of 3D printed prostheses. As these restorations might remain post-orthodontic treatment, reducing surface damage during removal is crucial to maintaining their appearance and functionality. Clinically, the results endorse the use of universal and air abrasion adhesives as an effective bonding method for clear aligner attachments on definitive 3D printed crowns, particularly for adult patients receiving orthodontic treatments involving prosthetic components.

## 5. Conclusions

Based on the limitations of this in vitro study, the following conclusions were reached.

- The tested 3D printed definitive resin (Crowntec) demonstrated a hybrid structure composed of an amorphous polymer matrix reinforced with approximately 33% inorganic fillers, as confirmed by EDS, XRD and TGA analyses.
- Among the evaluated protocols, airborne particle abrasion with 50 µm Al_2_O_3_ followed by the application of a universal adhesive (containing 10-MDP and silane) achieved the highest shear bond strength, exceeding clinically accepted thresholds for orthodontic retention.
- Polished, untreated resin surfaces exhibited inadequate adhesion for clinical use, highlighting the need for substrate pretreatment in aligner-based orthodontic workflows.

## Figures and Tables

**Table 1:** Materials used 3D printed composite resins for definitive restoration.

Material	Chemical Composition	Manufacturer
3D Printed composite resins for definitive restoration		
Crowntec^®^ (additively manufactured composite resin)	Esterification products of 4.4′-isopropylphenol, ethoxylated and 2-methylprop-2enoic acid, silanized dental glass, pyrogenic silica, initiator. Total content of inorganic fillers: 30–50 wt%	SAREMCO Dental AG
Surface Conditioning Methods		
Porcelain etchant (4% HFA)	Hydrofluoric Acid 5–10% Sodium Fluoride < 1	BISCO Inc.
Rhino (Al_2_O_3_)	Aluminum oxide particles White Alumina WA-200 (50 μm)	MDC Dental
Bonding agents		
Single bond universal adhesive (SBU)	MDP phosphate monomer, Dimethacrylate resins, HEMA, Vitrebond copolymer, filler, ethanol, water, initiators, silane.	3M^TM^ ESPE^TM^
Transbond XT Light Cure Adhesive Primer	Bisphenol a diglycidyl ether dimethacrylate, Triethylene glycol dimethacrylate (TEGDMA)	3M^TM^ Unitek^TM^
Composite resin		
3M Filtek^TM^ Z350 XT composite	Matrix: Bis-GMA, UDMA, Bis-EMA Filler: Silica, zirconia nanoparticles (20 µm) (72.5 wt%/55.9 vol%)	3M^TM^ ESPE^TM^

**Table 2:** Two-way ANOVA for the effects of surface treatment and adhesive system on shear bond strength (SBS).

	Type III Sum of Squares	df	Mean Square	*F*
**Surface Treatment**	564.9	2	282.45	125.41
**Adhesive**	167.45	1	167.45	74.35
**Surface Treatment x Adhesive Interaction**	18.86	2	9.43	4.19
**Error**	148.65	66	2.25	

**Table 3:** Pairwise comparisons of shear bond strength (SBS) values between treatment–adhesive combinations using the Games–Howell post hoc test. ΔMPa indicates the difference in the mean SBS between the groups. The *p* < 0.05 denote statistically significant differences.

Comparison	ΔMPa	*p*-Value
SBU (Air Abrasion)—TXT (Air Abrasion)	4.48	**<0.001**
SBU (Air Abrasion)—SBU (Etching)	6.28	**<0.001**
SBU (Air Abrasion)—SBU (Control)	7.59	**<0.001**
TXT (Air Abrasion)—TXT (Etching)	4.33	**<0.001**
TXT (Air Abrasion)—TXT (Control)	5.25	**<0.001**
SBU (Etching)—TXT (Etching)	2.53	**<0.001**
SBU (Control)—TXT (Control)	2.14	**<0.001**
SBU (Etching)—SBU (Control)	1.31	0.545
TXT (Etching)—TXT (Control)	0.92	0.759

## Data Availability

The original contributions presented in this study are included in the article. Further inquiries can be directed to the corresponding author.
